# Developmental Regulation of Nucleolus Size during *Drosophila* Eye Differentiation

**DOI:** 10.1371/journal.pone.0058266

**Published:** 2013-03-05

**Authors:** Nicholas E. Baker

**Affiliations:** Departments of Genetics, Ophthalmology and Visual Sciences, and Developmental and Molecular Biology, Albert Einstein College of Medicine, Bronx, New York, United States of America; University of Massachusetts Medical School, United States of America

## Abstract

When cell cycle withdrawal accompanies terminal differentiation, biosynthesis and cellular growth are likely to change also. In this study, nucleolus size was monitored during cell fate specification in the *Drosophila* eye imaginal disc using fibrillarin antibody labeling. Nucleolus size is an indicator of ribosome biogenesis and can correlate with cellular growth rate. Nucleolar size was reduced significantly during cell fate specification and differentiation, predominantly as eye disc cells entered a cell cycle arrest that preceded cell fate specification. This reduction in nucleolus size required Dpp and Hh signaling. A transient enlargement of the nucleolus accompanied cell division in the Second Mitotic Wave. Nucleoli continued to diminish in postmitotic cells following fate specification. These results suggest that cellular growth is regulated early in the transition from proliferating progenitor cells to terminal cell fate specification, contemporary with regulation of the cell cycle, and requiring the same extracellular signals.

## Introduction

The differentiation of multipotent progenitor cells into terminal cell types must involve a number of coordinated changes. Since different cell types have distinct proliferative properties (differentiated neurons, for example, are permanently post-mitotic), the determination of cell fate must be coordinated with cell cycle behavior. The end of proliferation is likely to be coordinated with changes in cellular growth, since doubling of cell components is necessary for mitotic cell populations to maintain cell size, but is not necessary for non-dividing cells. It is likely that changes in growth are further correlated with other aspects of cell physiology, including metabolism and energetics. Mechanisms should therefore exist to co-regulate multiple cellular processes during terminal differentiation.

The *Drosophila* eye imaginal disc, like many other developing tissues, grows rapidly and then becomes largely post-mitotic on terminal differentiation [1]. The spatial and temporal features of cell proliferation that accompany differentiation, as well as their control by extracellular signaling molecules, have been extensively studied [2], [3]. The eye imaginal disc is a favorable tissue to study changes that accompany terminal cell fate specification, because terminal cell fates are specified and postmitotic differentiation begins before pupariation complicates experimental access to the tissue, and because the progression of development is revealed spatially so that successive developmental stages are present simultaneously within each eye imaginal disc [4], [5]. The changes in the cell cycle that accompany eye differentiation, and their regulation, have been studied previously [1], [2], [3], [4]. Here, the changes and regulation of nucleolar size are addressed.

The nucleolus is the site of ribosomal RNA transcription and initial assembly of ribosomal subunits. Accordingly, nucleolar size is one simple albeit indirect measure of ribosome biogenesis and cellular growth, and is proportional to growth rate in cell lines and in tumor cells [6], [7]. In *Drosophila,* nucleoli are readily revealed by antibody labeling for the nucleolar protein fibrillarin, a rRNA 2′-O-methyltransferase that processes pre-ribosomal RNA [8], [9].

**Figure 1:**
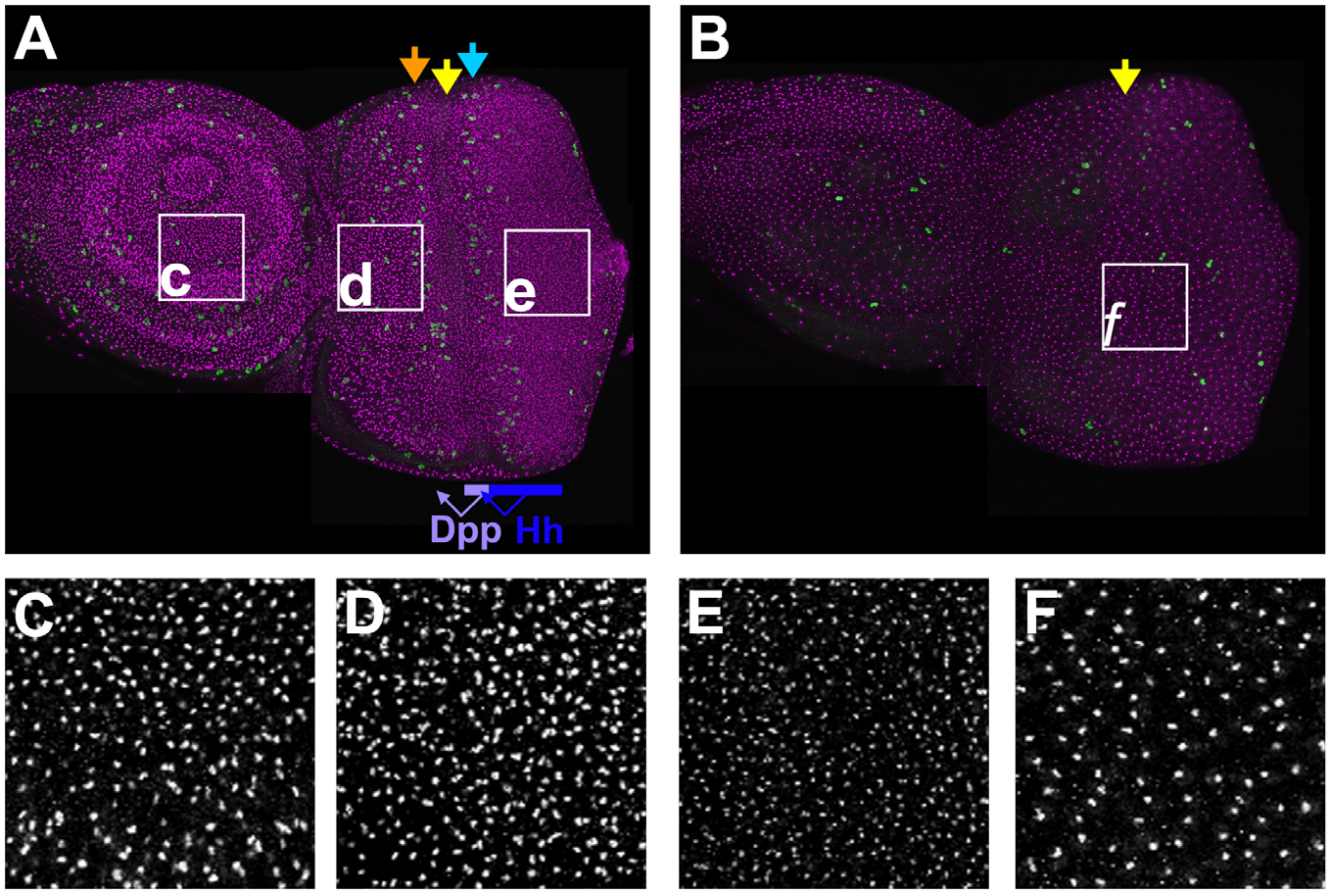
Nucleoli in the eye–antennal imaginal disc. A. Epithelial layer of the eye-antennal imaginal disc. Nucleoli labeled in magenta (anti-fibrillarin); mitotic figures labelled in green (anti-phosphoH3). Postmitotic fate specification begins within the morphogenetic furrow (yellow arrow), flanked by a First Mitotic Wave where unpatterned proliferation ends (orange arrow), and the Second Mitotic Wave where a subset of retinal progenitor cells divide (blue arrow). Blue bar indicates the approximate extent of Hedgehog (Hh) expression posterior to the furrow. Hedgehog induces Dpp within the furrow (lavender bar). Dpp and Hh together regulate differentiation and the cell cycle arrest that precedes the furrow (see text). B. Peripodial layer of the eye-antennal imaginal disc. Proliferation is largely unpatterned. C. Higher magnification of antennal region (box ‘c’ in panel A). Nucleoli were smaller in the cells of more distal regions (towards the top). D. Higher magnification of anterior eye region (box ‘d’ in panel A). Nucleoli resemble those from the antennal disc. E. Higher magnification of posterior eye region (box ‘e’ in panel A). Nucleoli are much smaller than in the antennal disc or anterior eye disc. Labeling intensity is also reduced (peak labeling is approx 0.6x the gray value of panel F). F. Higher magnification of the peripodial epithelium. Nucleoli are similar in size to the anterior eye disc and the antennal disc, but larger and more intense than nucleoli from the posterior eye region (compare panel E).

Cell fate specification begins during the third and final larval instar. A wave of differentiation moves anteriorly across the eye imaginal disc, starting from the posterior margin, until after two days the entire retinal field is differentiating (Figure 1A) [4]. A discernible groove called the ‘morphogenetic furrow’ moves anteriorly across the retinal field in concert with cell fate specification. Before differentiation begins, the eye imaginal disc consists of proliferating progenitor cells, as the eye imaginal disc grows ∼1000 fold from its primordial size after embryogenesis [1]. As the morphogenetic furrow moves across the disc, cells progressively arrest ahead of the furrow in G1 phase of the cell cycle (Figure 1A), about 17 cell diameters anterior to the first identified individual R8 photoreceptor precursors [10]. Although the five photoreceptor cells types that are specified within the morphogenetic furrow never return to the cell cycle, the remaining cells synchronously re-enter the cell cycle posterior to the morphogenetic furrow in a ‘Second Mitotic Wave’, dividing again before their fate specification and terminal arrest [1], [2], [3], [4]. The duration of the G1 that precedes the Second Mitotic Wave in these cells is estimated by different methods at 15–20 h [10] or 5–6 h [11].

The G1 arrest of eye cells depends on the secreted signals Dpp and Hh [2], [10]. These signals, which spread anteriorly from the morphogenetic furrow and from cells behind it (Figure 1A), are also responsible for the progressive initiation of differentiation and therefore for moving the morphogenetic furrow across the eye disc [12], [13], [14], [15]. Dpp is the most important signal for cell cycle arrest, but Hh brings about a delayed arrest even in cells that cannot transduce Dpp signals [10], [16]. Ectopic Dpp signaling is also sufficient for premature cell cycle arrest in a portion of the anterior eye disc [17], [18]. Although Hh was also suggested to play a role in the Second Mitotic Wave, it is now thought that this effect is indirect and that Notch signaling is responsible for the subset of cells that re-enter the cell cycle during the Second Mitotic Wave [10], [19]. This response to Notch signaling is balanced in differentiating cells by the opposing activity of EGF receptor pathway which promotes their cell cycle arrest as well as their cell fate specification [10], [19].

It was of interest to determine whether nucleolar size, an indirect indicator of ribosome biogenesis, decreased with larval eye disc differentiation, when such changes would occur, and how they might be regulated. It would be useful to assess how changes in nucleolar size correlated with particular cell cycle arrest or cell proliferation events, and how any such coordination might be achieved. Accordingly, substantial changes in nucleolar size in the eye disc that accompany the transition from multipotent progenitors to differentiating retinal cells are described.

## Results

### Changes in Nucleolar Size during Eye Disc Development

Nucleoli can be visualized through antibody labeling to detect the fibrillarin component of the nucleolar snRNP particle [20]. Fibrillarin was labeled in whole eye antennal disc complexes and examined by confocal microscopy to estimate nucleolar size. Specifically, we measured absolute nucleolar size as cross-sectional areas in confocal sections, without regard to cell size, nuclear size, or labeling intensity (see Methods for details). Nucleolar size seemed similar in antennal discs, peripodial membrane, and anterior regions of the eye disc (Figures 1A–D and 1F), although nucleoli within the antennal disc were somewhat smaller in distal cells than proximal cells (Figure 1C). In contrast to these proliferative tissue regions, nucleoli were visibly much smaller, and labeled less intensely, in the differentiating, predominantly postmitotic portion of the posterior eye disc, consistent with reduced ribosome biogenesis in these non-dividing cells (Figures 1A and 1E).

**Figure 2:**
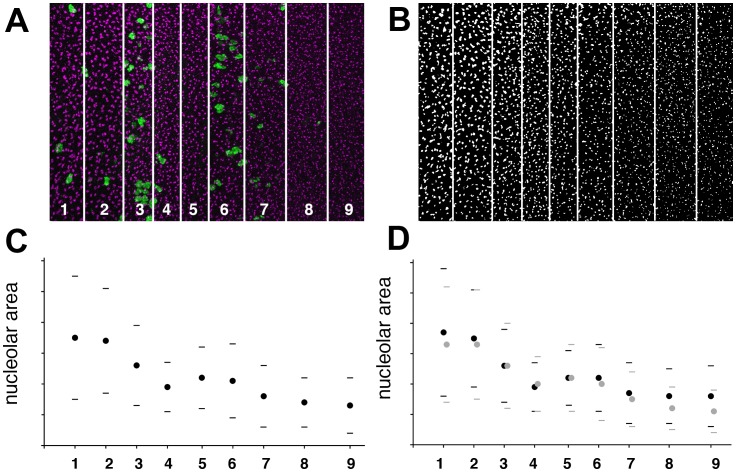
Timecourse of changes in nucleolus size. A. High resolution micrograph of the eye imaginal disc, divided into nine zones from anterior to posterior, according to mitotic behavior (see text for details). Nucleoli labeled in magenta (anti-fibrillarin); mitotic figures labelled in green (anti-phosphoH3). B. Nucleolar labeling from panel A, z-projected from apical to basal within the disc epithelium and digitally processed for automated measurement (see methods). C. Nucleolus cross-section in pixels. Mean±1 standard deviation shown for each zone. D. Nucleolus cross-section in pixels. Mean±1 standard deviation shown. Within each zone, nucleoli from separated 3–5 μ deep apical or basal layers of the disc epithelium were analyzed separately (basal nucleoli: black circles; apical nucleoli, grey circles). These data were pooled in panel C.

To define a time-course, cross-sectional areas of individual nucleoli were recorded across the anterior-posterior axis of eye discs (Figure 2). The known cell cycle transitions were used to define nine zones that could be detected by double-labeling for cell cycle markers (Figure 2). Zones 1 and 2 lay within the anterior, undifferentiated eye disc. Zone 3 included the last mitotic figures before the G1-phase cell cycle arrest that precedes the morphogenetic furrow. The frequency of mitotic figures is often highest in the Zone 3 region, which is called the ‘First Mitotic Wave’ [3]. Zones 4 and 5 occupied the anterior and posterior halves of the zone arrested in G1-phase. The morphogenetic furrow overlaps Zone 5. Zone 6 corresponded to the peak of the Second Mitotic Wave cell divisions that follows immediately posterior to the morphogenetic furrow. The differentiating precursors of the R2, R3, R4, R5, and R8 cells remain arrested in the Second Mitotic Wave but all the other cells perform another cell cycle [4]. Zones 7,8, and 9 corresponded to progressively later portions of the differentiating retina, during which fewer and fewer mitotic figures are seen and the number of postmitotic cells approaches 100% [4], [21].

Mean nucleolar cross-section was nearly 65% smaller in zone 9 near the posterior disc margin than in zone 1 in the anterior. The results of plotting median nucleolar size were similar (not shown). Figure 2C shows that the major decrease in nucleolar cross-section occurred between Zones 2 and Zone 4. This centers on Zone 3, corresponding to the first mitotic wave and onset of G1 arrest ahead of the furrow. A further, less pronounced reduction in nucleoli affected more mature, differentiating, postmitotic cells posterior to the morphogenetic furrow, from Zones 7–9.

### Nucleolus Size in the Second Mitotic Wave

There often appeared to be a temporary halt or small reversal of the trend towards smaller nucleoli around zones 5–6, which could be related to the Second Mitotic Wave (Figure 2C). Which cells will enter the Second Mitotic Wave is probably determined, and these cells exit G1 and enter S-phase, during Zone 5; the wave of mitosis itself is centered on Zone 6 [23](Figure 2). To see whether nucleolus size differed in the specific subset of Second Mitotic Wave cells, nuclear position in the apical-basal axis was used to distinguish cells that will divide in the Second Mitotic Wave. Nuclei of differentiating ommatidial cells rise near the apical surface of the disc epithelium as their fates are specified, and although they subsequently fall somewhat, they never again drop to the basal level of the nuclei of cells that have remained unspecified [22]. As a consequence, the nuclei of cells destined not to divide in the Second Mitotic Wave because they have already been specified are more apical, while the nuclei of cells that have not been specified and participate in the Second Mitotic Wave occupy more basal positions. The size of the nucleoli from basal and apical nuclei of the same disc were not distinguishable statistically during the Second Mitotic Wave (Figure 2D) but see the following paragraph for a different analysis. It was noted, however, that the late reduction in nucleolar size that occurs after Zone 7 predominantly affected differentiating ommatidial cells with more apical nuclei, whereas the nucleoli of unspecified cells with basal nuclei changed less after the Second Mitotic Wave (Figure 2D).

**Figure 3:**
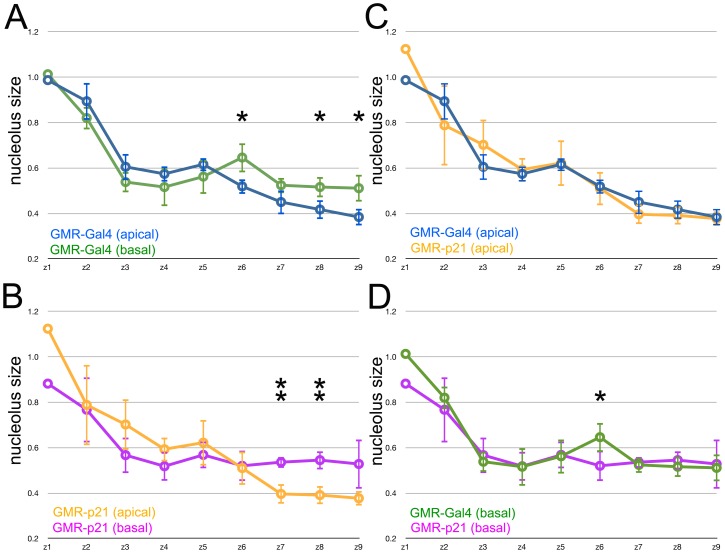
Effect of p21^Cip1/WAF1^ expression on nucleolus size. Nucleolus size over time. Data shown are mean and standard deviations of the average nucleolus size from each Zone, normalized to Zone 1 from the same eye disc. Statistical significance between samples: *, p<0.05; **, p<0.01; ***, p<0.001. A. Blue - cells with apical nuclei in GMR-Gal4/+; Green - cells with basal nuclei in GMR-Gal4/+. Nucleoli from unspecified cells with basal nuclei were significantly larger in Zones 6, 8 & 9. B. Orange - cells with apical nuclei in *GMR-p21*; Magenta - cells with basal nuclei in *GMR-p21.* Nucleoli from unspecified cells with basal nuclei were significantly larger in Zones 7 & 8. Note that nucleolar sizes did not differ in Zone 6. C. Comparison of apical nucleoli from GMR-Gal4/+ (blue) and GMR-p21 (orange). No significant differences were noted. D. Comparison of basal nucleoli from GMR-Gal4/+ (green) and GMR-p21 (magenta). Basal nucleoli from GMR-p21 were significantly smaller in Zone 6.

In order to assess the Second Mitotic Wave further, this cell cycle was prevented by overexpressing human p21^Cip1/WAF1^ posterior to the morphogenetic furrow [23]. To compare nucleoli between genotypes, the mean size of apical and basal nucleoli from each zone was determined for each of multiple discs of each genotype, normalized to the nucleolus size in zone 1 of each disc, and the means from each genotype compared (Figure 3). This led to two conclusions. First, in the control genotype (*GMR-Gal4*/+) the mean size of apical and basal nuclei was significantly different in zone 6, being larger in the basal nuclei that participate in the Second Mitotic Wave, and smaller in the apical nuclei of cells that are differentiating not dividing at this stage (Figure 3A). This difference had not been significant statistically in the nucleoli from a single disc because of the scatter in the measurements (Figure 2D), but was significant when mean data from multiple discs were analyzed (Figure 3A). Secondly, no such difference was observed in zone 6 of *GMR-p21* eye discs (Figure 3B). When the size of nucleoli of apical, differentiating nuclei from control and *GMR-p21* discs was compared, no significant differences were observed (Figure 3C). By contrast, nucleoli of basal nuclei were significantly larger in zone 6 of controls than in the *GMR-p21* genotype that lacks the Second Mitotic Wave (Figure 3D). These data suggest that the Second Mitotic Wave was associated with larger nucleoli in the dividing cell population with basal nuclei, and that p21 expression prevented this.

In Zones 7–9 posterior to the Second Mitotic Wave, the nucleoli of differentiating cells from GMR-p21 discs were indistinguishable from the controls at these stages, as were the nucleoli of the progenitor pool with basal nuclei (Figures 3C,D). As would be expected therefore, the increasingly smaller nucleolar size in differentiating cells was significant statistically in both control and GMR-p21 genotypes (Figure 3A,B). Ahead of the morphogenetic furrow, in Zones 1–4, nucleoli from apical and basal nuclei were indistinguishable, and the same in GMR-p21 and controls (Figure 3). This was expected, as progenitor cells ahead of the furrow are thought to be equivalent developmentally, their nuclear position does not correlate with fate, and the GMR promoter is not active in them.

### Stimulating Cell Division

To explore the effects on nucleolar size, extra cell cycles were driven using over- expression of Cyclins. Previous studies have distinguished between the effects of over-expressing Cyclin E and the effects of over-expressing Cyclin D along with its partner Cdk4. The conclusion has been that Cyclin D and its partner Cdk4 primarily drive cellular growth, which in turn accelerates S-phase entry in cell populations capable of mitosis, whereas Cyclin E and its partner Cdk2 promote cell cycle entry more directly, bypassing come or all cellular growth [24], [25], [26], [27].

**Figure 4:**
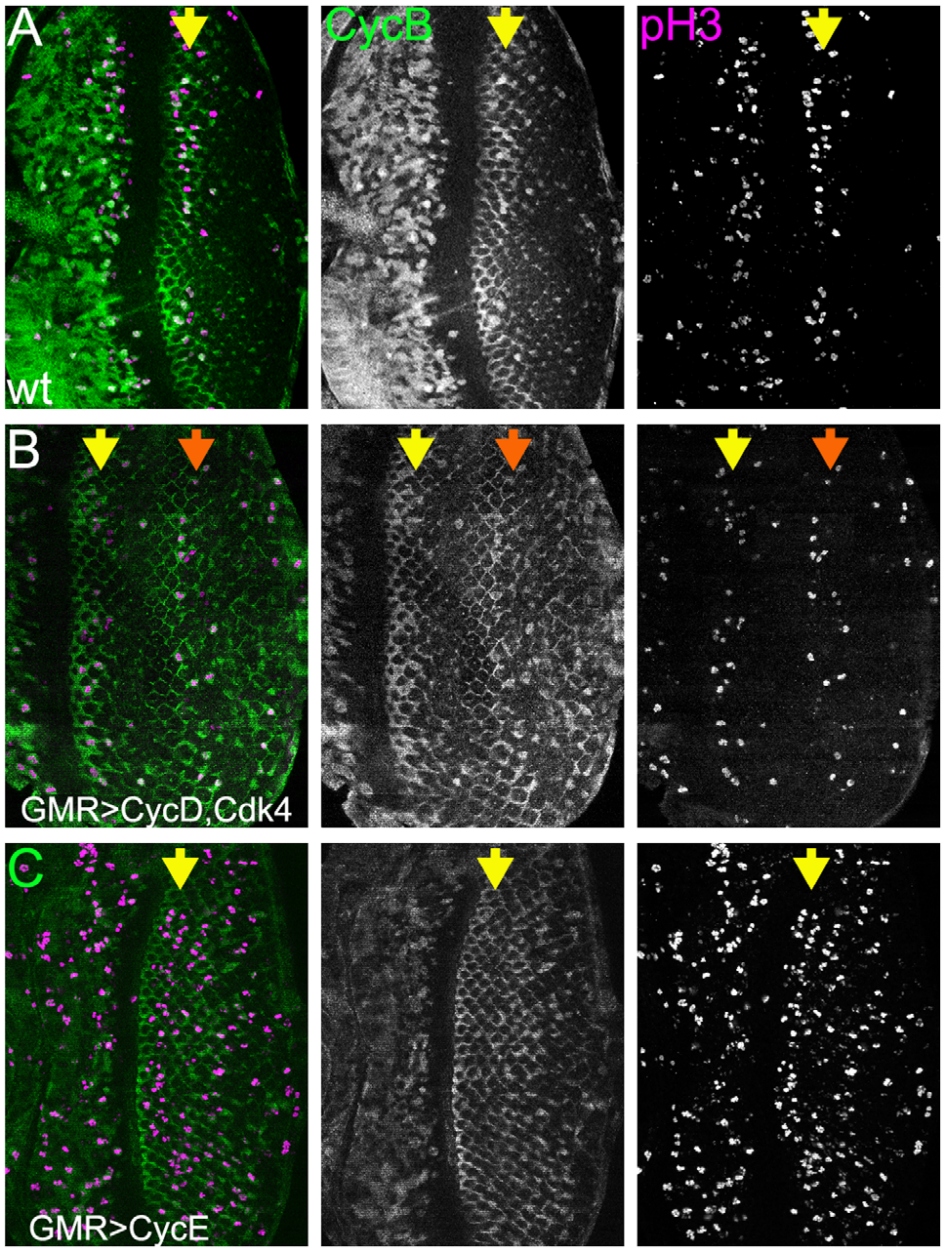
Proliferation induced by Cyclin overexpression. All panels show Cyclin B protein in green as a marker for cells between S-phase and mitotic metaphase, and pH3 in magenta as a marker for mitotic cells. The yellow arrow indicates the Second Mitotic Wave in all figures. A) wild type. Most Second Mitotic Wave mitosis occurs in a band close to the morphogenetic furrow. B) GMR-GAL4, UAS-CycD, UAS-Cdk4. The Second Mitotic Wave is followed by a fairly synchronous additional cell cycle approximately 8 columns more posteriorly. The peak of mitosis in this “Third Mitotic Wave” is indicated by the orange arrow. The Second and Third Mitotic Waves are clearly separated by a zone where most cells are in G1 phase and lack Cyclin B. C) GMR-GAL4, UAS-CycE. The Second Mitotic Wave is followed by generalized and apparently unsynchronized proliferation of interommatidial cells.

When either Cyclin E or Cyclin D and Cdk4 were over-expressed posterior to the morphogenetic furrow using the *GMR-Gal4* driver, ectopic cell cycles resulted in the population of eye disc cells that was not yet differentiated, but very rarely in the differentiating photoreceptor cells that were already postmitotic (Figure 4 and data not shown). There was a difference in the timing of cell cycle responses, revealed both by labeling for mitotic figures and by labeling for cell cycle entry using anti-Cyclin B. Cyclin B protein accumulates in cells after the start of S-phase and is destroyed at mitotic metaphase [28], [29], [30], [31]. Whereas extra cell cycle entry and mitotic figures appeared randomly distributed amongst unspecified cells over-expressing Cyclin E (Figure 4C), Cyclin D and Cdk4 drove a more synchronous wave of extra cell cycles concentrated around a particular location posterior to the normal Second Mitotic Wave (Figure 4B). This distribution could be consistent with the proposed effects of Cyclin D and Cdk4 on cellular growth. If extra growth begins synchronously when GMR-Gal4 is activated in the morphogenetic furrow, growing cells are likely to achieve a size requiring cell cycle entry and division at roughly the same time, resulting in a distinct ‘third mitotic wave’. This was not seen with ectopic Cyclin E. We have even observed an additional ‘fourth mitotic wave’ in eye discs overexpressing the p110 subunit of Phospho-Inositol-3′ Kinase (data not shown), another regulator of cellular growth [32]. Since these observations were consistent with previous conclusions about the respective properties of Cyclin D and Cyclin E [24], [25], [26], [27], we expected that Cyclin D and Cdk4 might enhance nucleolar size in eye discs, and that Cyclin E might drive cell division without affecting nucleoli.

**Figure 5:**
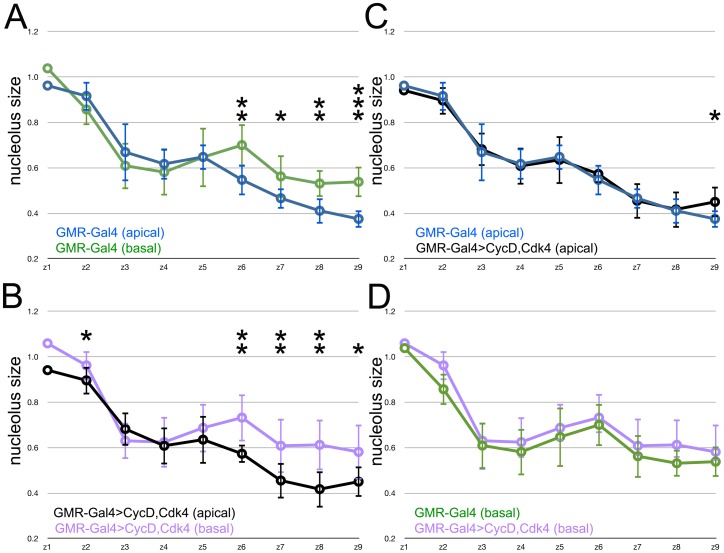
Effect of Cyclin D/Cdk4 expression on nucleolus size. Nucleolous size over time. Data shown are mean and standard deviations of the average nucleolus size from each Zone, normalized to Zone 1 from the same eye disc. Statistical significance between samples: *, p<0.05; **, p<0.01; ***, p<0.001. A. Blue - cells with apical nuclei in GMR-Gal4/+; Green - cells with basal nuclei in GMR-Gal4/+. Nucleoli from unspecified cells with basal nuclei were significantly larger in Zones 6–9. B. Black - cells with apical nuclei in GMR-Gal4 UAS-CycD UAS-Cdk4/+; Mauve - cells with basal nuclei in GMR-Gal4 UAS-CycD UAS-Cdk4/+. Nucleoli from unspecified cells with basal nuclei were significantly larger in Zones 6–9. The significant difference in Zone 2 is hard to interpret since GMR-Gal4 is not active there. C. Comparison of apical nucleoli from GMR-Gal4/+ (blue) and GMR-Gal4 UAS-CycD UAS-Cdk4/+ (black). No significant differences were noted until Zone 9. D. Comparison of basal nucleoli from GMR-Gal4/+ (green) and GMR-Gal4 UAS-CycD UAS-Cdk4/+ (mauve). No significant differences were noted.

When the mean size of nucleoli was examined after ectopic expression of Cyclin D/Cdk4, very little effect was seen (Figure 5). Similar to the control, nucleoli from the basal nuclei of undifferentiated cells were larger than their counterparts from the apical nuclei of differentiating cells from column 6 onwards (Figure 5A,B). CycD and Cdk4 did not increase the size of nucleoli in differentiating cells in most of the imaginal disc, but it is possible that an increase is becoming apparent in Zone 9 (Figure 5C). The nucleoli of undifferentiated eye disc cells with basal nuclei, ie the cells that undergo extra cell cycles, were not statistically different from controls at any stage (Figure 5D).

**Figure 6:**
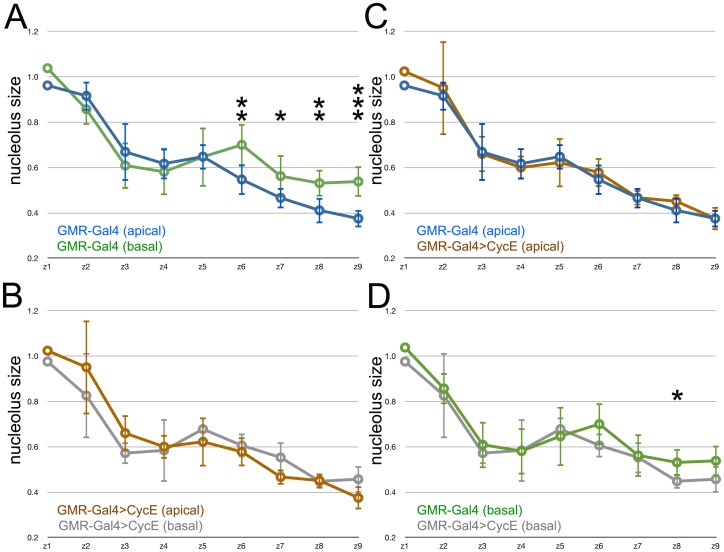
Effect of Cyclin E expression on nucleolus size. Nucleolous size over time. Data shown are mean and standard deviations of the average nucleolus size from each Zone, normalized to Zone 1 from the same eye disc. Statistical significance between samples: *, p<0.05; **, p<0.01; ***, p<0.001. A. Blue - cells with apical nuclei in GMR-Gal4/+; Green - cells with basal nuclei in GMR-Gal4/+ (same data as in Figure 5A). Nucleoli from unspecified cells with basal nuclei were significantly larger in Zones 6–9. B. Brown - cells with apical nuclei in GMR-Gal4 UAS-CycE/+; Gray - cells with basal nuclei in GMR-Gal4 UAS-CycD UAS-CycE/+. No significant differences were seen between nucleoli from unspecified cells with basal nuclei and nucleoli from differentiating cells with apical nuclei. C. Comparison of apical nucleoli from GMR-Gal4/+ (blue) and GMR-Gal4 UAS-CycE/+ (brown). No significant differences were noted. D. Comparison of basal nucleoli from GMR-Gal4/+ (green) and GMR-Gal4 UAS-CycE/+ (gray). From Zone 6 onwards nucleoli were smaller in GMR-Gal4 UAS-CycE/+ and this was statistically significant in Zone 8.

The results from over-expression of Cyclin E were slightly different. Cyclin E appeared to suppress the differences between differentiating and unspecified cells (Figure 6A,B). The unspecified cells with basal nuclei did not have larger nucleoli in Zone 6, and the differentiating cells with apical nuclei did not have smaller nculeoli in Zones 7–9 (Figure 6A,B). Compared to the control genotype, the nucleoli of differentiating cells with apical nuclei were were indistinguishable (Figure 6C). The nucleoli of unspecified cells with basal nuclei tended to be smaller than in the control genotype, although this was only sometimes significant statistically (Figure 6D).

### Regulation of Nucleolus Size Requires Dpp and Hh Signaling Pathways

**Figure 7:**
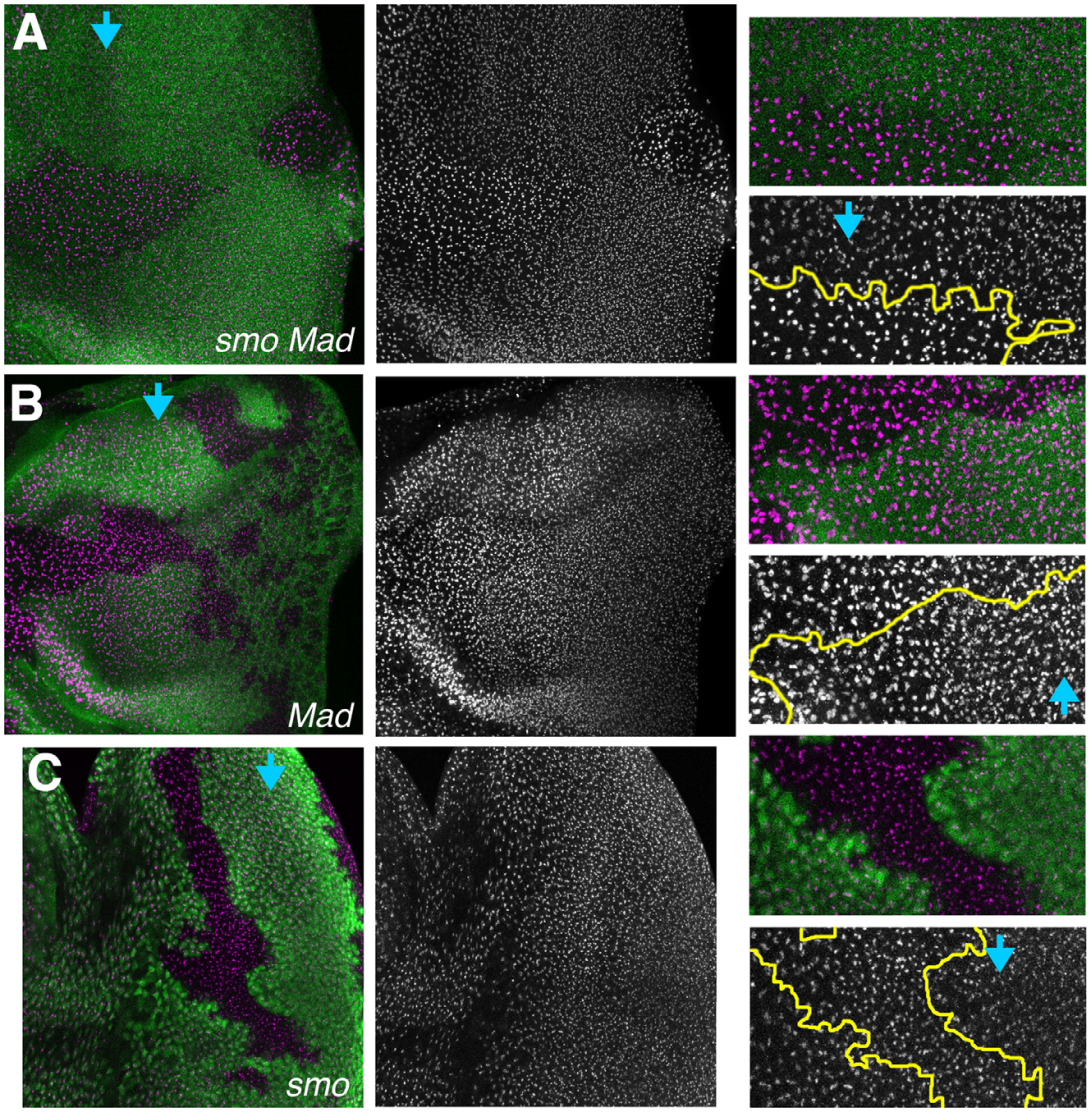
Requirement for Dpp and Hh signaling in nucleolar size. A. Eye imaginal disc labelled for nucleoli (magenta). Clones mutant for *smo* and *Mad* lack beta-galactosidase expression (green). Blue arrow shows the onset of fate specification in the morphogenetic furrow. Nucleoli remain the same size within *smo Mad* clones. Outside the clone nucleoli become progressively smaller. Nucleoli mutant for *smo* and *Mad* also appear more intensely labelled. Fibrillarin channel and enlargements shown to the right. The distinction is evident posterior to the furrow in the enlarged panels. B. Clones mutant for *Mad* lack beta-galactosidase expression (green). Nucleoli mutant for *Mad* shrink at the same time as in wild type cells. Fibrillarin channel and enlargements shown to the right. C. Clones mutant for *smo* lack beta-galactosidase expression (green). Nucleoli mutant for *smo* shrink at the same time as in wild type cells. Fibrillarin channel and enlargements shown to the right. In panels A–C, fibrillarin labeling is shown as a maximum projection of multiple layers, because nucleoli occupy varying positions in the z-axis. Beta-galactosidase labeling is shown for only a single, central, z-plane, so that parts of the negatively-marked clones were not obscured. To accurately determine the genotype of each nucleolus, beta-galactosidase and fibrillarin labeling must be examined for each confocal plane (not shown).

Fibrillarin labeling was next performed in mutant genotypes to determine whether extracellular signaling pathways were required to regulate nucleoli during eye development. Hh and Dpp signaling are the main signals driving the progression of differentiation across the eye disc, and already known to affect the cell cycle (Figure 1A) [2], [5], [10], [14], [15]. Figure 7A shows an eye disc containing clones of cells mutant for both *smo* and *Mad*, essential components for signal transduction in response to Hh and Dpp, respectively. The mutant cells revealed a failure to reduce nucleolar size from anterior to posterior. In the mutant clones, nucleolar size at all locations remained as large as in unspecified progenitor cells ahead of the morphogenetic furrow, even in cells in zone 9 near the posterior margin of the eye disc, and the labeling was intense. These effects appeared cell-autonomous. Clones of cells individually mutant for one or other of *smo* and *Mad* were also examined (Figure 7B,C). Such single mutant cells had nucleoli that appeared similar to wild type cells of comparable developmental stage outside the clones. These data indicate that nucleolus size is regulated by Hh and Dpp signaling as the morphogenetic furrow progresses. Hh and Dpp were required redundantly. Whether ectopic Dpp or Hh signaling ahead of the furrow would be sufficient to reduce the size of nucleoli precociously was not examined.

## Discussion

Since postmitotic cells do not need to grow in order to maintain cell size, it is to be expected that cellular growth rate might decline when terminal cell fates are specified and differentiation begins. Here, we focus on the transition that occurs in the eye disc between proliferating progenitor cells and specified, post-mitotic cells. The *Drosophila* eye imaginal disc is convenient for such studies because a wave of differentiation sweeps across the tissue so that each preparation displays successive developmental stages across the anterior-posterior axis, and these can be compared directly. Of course postmitotic cells may also change size as part of specific differentiation processes, for example *Drosophila* retinal cells enlarge in the pupa, a later stage that we have not examined [1].

The main finding is that nucleolus size, an indicator of ribosome biogenesis, is much reduced behind the furrow in comparison to proliferative cells ahead of the furrow, or compared to the proliferating antennal disc or peripodial epithelium. Much of the reduction in nucleolus size occurs at the same time that cells enter a prolonged G1 arrest ahead of the morphogenetic furrow, and is a cell-autonomous response to Hh and Dpp, the same signals that regulate the G1 arrest (Figure 7) [10]. The Second Mitotic Wave, a synchronous cell cycle that affects only the subpopulation of cells that are still unspecified just behind the morphogenetic furrow, is associated with a small but statistically significant size increase in the nucleoli only of this cell population, and which is prevented by expression of human p21, which also prevents cell cycle entry (Figure 3) [23]. These findings provide evidence that ribosome biogenesis, at least one component of cellular growth, is reduced with terminal differentiation and that this coordination is mediated by Hh and Dpp signaling.

Although mutant cells unable to respond to Hh and Dpp continue to progress through the cell cycle, previous measurements of mitotic figures and of S-phase DNA synthesis suggest that they do so rather slowly, and it is not clear that the mutant cells increase in size [10], [11]. It would be interesting to determine whether maintaining nucleolar size is sufficient to maintain cellular growth, as nucleolar size may not be the only factor limiting cell cycle progression as the morphogenetic furrow approaches. Other indicators of protein synthesis that also could be assessed would be the nascent transcription of rRNA or tRNA, or the distribution of mature ribosomes. Cellular growth also depends on biosynthesis of other molecules besides proteins.

Coordination of ribosome biogenesis with the cell cycle raises the question of whether one regulates the other. If cell cycle progression feeds forward on to growth, this might explain how human p21 interferes with the growth of nucleoli of cells that should enter the Second Mitotic Wave. Preventing cell cycle arrest by forced expression of cyclins did not lead to a clear conclusion. Levels of Cyclins sufficient to cause an extra round of cell divisions posterior to the morphogenetic did not increase nucleolar size. It is possible that cell cycle progression is necessary to increase nucleolar size but not sufficient, or that human p21 interferes with ribosome biogenesis independently of blocking S-phase entry.

There is also abundant evidence for the coupling of cellular growth to cell cycle progression, such that cell growth is sometimes considered the first step in the cell cycle, and its absence is a key distinction between G0 and cycling cells [33], [34]. Sensitivity of cell cycle protein translation to growth conditions is one mechanism that has been proposed [35]–[36]. Cell cycle progression and growth may also be co-regulated in a parallel fashion, for example as common transcriptional targets of the DNA Replication Related Element binding Factor (DREF) [37]. Previous studies have concluded that Cyclin D and Cdk4 promote cellular growth as a major part of their effect because ectopic cell division driven by Cyclin D and Cdk4 over-expression does not reduce cell size, and because these proteins can increase the size of postmitotic cells [24], [25]. The only indication of a possible effect on ribosome biogenesis in our experiments was in postmitotic, differentiating cells at the back of the disc (Figure 5C), however, not in the cells that divide in response to CyclinD and Cdk4. It is possible that Cyclin D and Cdk4 affect cellular growth through other pathways such as mitochondrial biogenesis [38], [39]. Another possibility is that division of the cell’s components at mitosis might transiently reduce the size of nucleoli, masking any increase due to Cyclin D/cdk4, and potentially reducing the size of nucleoli in cells over-expressing Cyclin E (Figure 6).

A key target of Dpp and Hh with regard to eye differentiation is thought to be the proneural gene *atonal*, which is required to specify the first photoreceptor cells but does not contribute to cell cycle arrest ahead of the furrow [28], [40], [41]. In addition it is uncertain whether *ato* is regulated directly by the Mad and Ci transcription factors that are the targets of Dpp and Hh signal transduction [42]. Recently, it has been suggested that cell cycle arrest depends on repressing the Meis-family protooncogene homolog *homothorax* [43]. Another model posits that it is changes in Dpp signaling level, either in space or in time, that are required for proliferation [44], [45]. We hypothesize that differentiation, cell cycle arrest, and the attenuation of cellular growth are somewhat independent processes, coordinated by each sharing regulation from Hh and Dpp signaling in the eye. As has been noted before regarding the regulation of both differentiation and the cell cycle by the same extracellular signals, since so much developmental signaling is mediated by a small number of cell-cell signaling pathways, coordination (or antagonism) between developmental processes can be a natural consequence of regulation by common extracellular signals [46].

It would be no surprise if changes in ribosome biogenesis were accompanied by changes in other processes such as energy generation and protein turnover. Consistent with this idea, genome-wide studies point to large, antagonistic human gene networks whose expression changes as proliferation gives way to differentiation [47], and indicate that expression of a significant fraction of eukaryote genomes may be regulated by growth rate [48]. These studies have been based on changes during aging or in nutrient availability, however, and it remains to be seen how regulation occurs during developmental patterning, when cellular growth rate can even differ within the same tissue at the same time, as seems to be the case in the eye imaginal disc.

## Materials and Methods

### Tissue Staining and Immunofluorescence

Primary antibodies used were rabbit anti-ß-Galactosidase (Cappel), mouse and rabbit anti-GFP antibodies (Invitrogen #A11120 and A11122); rabbit anti-phosphoHistone3 (Cell Signaling Technology #9701), mouse monoclonal anti-fibrillarin (38F3, Abcam #ab4566), and mouse monoclonal anti-Cyclin B (F2F4). Secondary antibodies were Cy2- and Cy3-conjugates from Jackson Immunoresearch. Labeling of eye discs was performed as described [37]. Preparations were examined on BioRad Radiance 2000 or Leica SP2 Confocal microscopes. To measure cross-sectional areas of nucleoli, confocal sections were projected using maximum projection. Nucleolar sizes were measured without human selection: experimental and digital noise was minimized by applying a 1.5 pixel Median Filter in NIH ImageJ software v1.36b (Figure 2), or using Despeckle in v1.44j (Figures 3, 5, 6), nucleoli isolated by applying the Thresholds adjustment, and cross-sectional areas measured by the Analyze Particles function. For the separation of cell populations with apical and basal nuclei, 3–5 one-micron confocal sections within the basal or apical portions of the epithelial layer were maximum-projected and analyzed. Data plotted in Figure 3C reflect the pooled data. Projecting nucleolar signal through the entire epithelium does not change the overall findings, but results in a small increase in apparent mean nucleolar area (but not median), and large increase in standard deviations. We think this is due to overlapping projection of nearby nucleoli at different locations in the z-axis generating a small class of anomalously large objects. For consistency, nucleoli from cells with more apical or basal nuclei were also analyzed separately in the anterior eye disc regions where nuclear position does not correlate with fate. Statistical significance was determined using two-tailed t-tests assuming equal variances, unless unequal variances were indicated by F-tests.

### Mitotic Clone Induction

Clones of cells mutant for genes were obtained by the FLP-mediated mitotic recombination technique [49], [50]. Homozygous mutant cells were identified through the absence of transgene encoded markers arm-βgal or ubiGFP [51]. *Mothers against dpp (Mad)* clones were obtained in hsF; *Mad^1^* ^2^ FRT40/M(2)24F [*armLacZ*] FRT40. *Mad^1^* ^2^ is a null allele that lacks the N-terminal sequences for phosphorylation by BMP family receptors [52], [53]. *smo* clones were obtained in hsF; *Mad^1^* ^2^ FRT40/ubiGFP FRT40. *smo^3^* is a missense allele that is genetically null [54]. *smo Mad c*lones were obtained in hsF; *smo^3^ Mad^1^* ^2^ FRT40/M(2)24F [*armLacZ*] FRT40.
